# Association between Exposure to Extreme Temperature and Injury at the Workplace

**DOI:** 10.3390/ijerph16244955

**Published:** 2019-12-06

**Authors:** Junhyeong Lee, Wanhyung Lee, Won-Jun Choi, Seong-Kyu Kang, Seunghon Ham

**Affiliations:** Department of Occupational and Environmental Medicine, Gil Medical Center, Gachon University College of Medicine, Incheon 21565, Korea

**Keywords:** high temperature, low temperature, personal protective equipment, occupational injury, Korean Working Condition Survey

## Abstract

Exposure to extreme temperature is a critical occupational risk factor. This study aimed to investigate the association between exposure to extreme temperatures and injury at the workplace using data from 92,238 workers (46,175 male and 46,063 female) from the 2014 and 2017 Korean Working Condition Survey. Exposure to extremely high or low temperatures, injury experiences, and personal protective equipment (PPE) wearing behavior were investigated using a questionnaire. Logistic regression analyses were performed to investigate the association between exposure to extreme temperature and injury experience. The association between injury experience and PPE wearing behavior was analyzed for each exposure group. After adjusting for individual and occupational factors, the odds ratios (ORs) for injury experience were 2.06 (95% confidence interval (CI): 1.78–2.38) and 1.64 (95% CI: 1.44–1.85) in both high and low temperature exposure groups, respectively, and 1.45 (95% CI: 1.15–1.83) for those not wearing PPE when exposed to high temperature. There was no significant association shown with wearing PPE and injury experience in the low temperature exposure group. Exposure to extreme temperature tended to increase the risk of injury, and was higher in workers not wearing PPE in high temperature. PPE that can be worn comfortably in high temperature is needed to prevent occupational injury.

## 1. Introduction

Exposure to extremely high or low temperatures can cause adverse health effects. Global warming is evident, with a global temperature increase of 0.85 °C between 1880 and 2012, and a forecasted increase of 3 °C by 2100 [1,2,3]. Moreover, the frequency of abnormal temperatures, such as heat waves and extreme cold weather, is increasing. In extreme environments, the incidences of cardiovascular disease, heat-related illnesses and frostbite increases [4,5,6,7,8,9]. The increase in heat wave frequency is also associated with mortality [10].

Both high and low temperature exposure can cause occupational injury [11,12]. Several epidemiological studies have reported significant increases in the incidences of injury in steelmaking [13], tile production processes [14], construction [15], and agriculture [16,17], because of high temperature exposure. Meanwhile, in the mining industry, injuries are increasing because of low temperature exposure [18].

In general, occupational injury caused by temperature is known to draw a U-shaped curve based on a 20 °C threshold [19]. According to a previous study [20], occupational injury increased by 0.2% when there was a 1 °C increase in temperature between 14.2 °C and 37.7 °C. A Canadian study has reported an increase in the accident incidence rate of 1.002 (95% confidence interval (CI) = 1.002–1.003) when outdoor temperatures rose by 1 °C during the summer in 16 areas across Quebec. Another study reported a 2.3% increase in the accident incidence rate, for each 1 °C decrease from −0.8 °C or below [21].

Occupational injury refers to the physical damage caused by an accident during a job performance, resulting in primary health problems, work delay and sick leave, as well as significant losses for workers and business owners alike [22]. Several studies have analyzed the correlation between high and low temperature exposure and the occurrence of injury [9,10,11,12,13,14,15,16,17,18,19,20,21,23,24]. However, most studies have often represented specific regions, or targeted specific jobs, such as agriculture, mining, and construction, even in the very countries where the study was conducted. These works were limited, as they failed to consider other covariates that could have caused the injuries.

In addition, there are reports that the characteristics of PPE, which inhibits body temperature control and evaporation of sweat, can cause heat-related illness at lower temperatures in certain professions, such as firefighters [17,25]. PPE, however, serves to protect workers from various harmful factors, preventing occupational injury, including serious trauma. There is no epidemiological study that has correlated PPE to occupational injury in extreme environments.

The present study aimed to identify the correlation between high and low temperature exposure, and occupational injury experience, using data from the Korean Working Conditions Survey (KWCS), which reflected the characteristics of workers in Korea, and analyzed the effects between PPE wearing behavior and occupational injury, when exposed to high and low temperatures.

## 2. Materials and Methods 

### 2.1. Study Design and Participants

The fourth (2014) and fifth (2017) Korean Working Conditions Surveys (KWCS) were conducted by the Korea Occupational Safety and Health Agency. The KWCS provides basic data that can be analyzed to determine the impact of variables on an employee’s health, including sex, age, job classification, and workplace environments, and is conducted by referring to the European Working Conditions Survey for safety and health policy establishment. The questionnaire-based survey includes the active working population of Koreans aged 15 years old and older, as well as individuals who were either employees or self-employed at the time of the interview. The basic sample design is a multistage, random sampling method based on the Population and Housing Census. All respondents agreed to participate in further scientific research, and were assigned random participant numbers to protect their anonymity.

**Figure 1:**
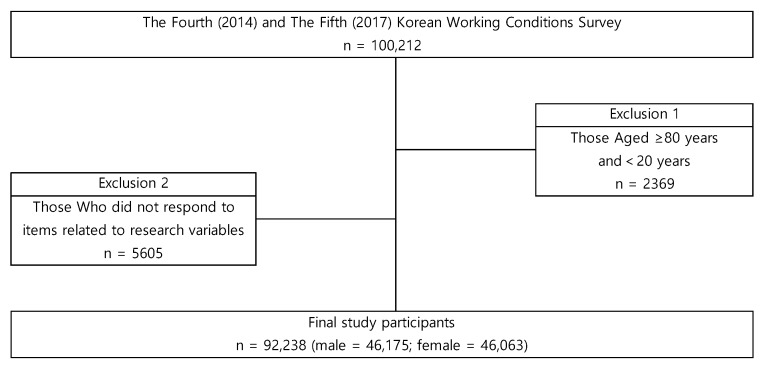
Schematic diagram depicting study population.

In the fourth and fifth KWCS, 50,007 and 50,205 workers participated, respectively. We merged the two data sets to ensure reliability by increasing the sample size. A total of 100,212 working individuals were surveyed. In an attempt to represent the general working population, 2369 participants older than 79 years old, and younger than 20 years old, were excluded from the final sample. We also excluded 5605 participants who did not respond to any of the questions on the research variables used in this study. Thus, 92,238 participants who answered all of the research variables were included in this study (Figure 1).

### 2.2. Main Variables

High and low temperature exposures were examined using a self-reported questionnaire. Exposure was assessed via a response to the question: “Please tell me, using the following scale, are you exposed at work to…?” Further questions for the participants went as follows: “In your workplace, are you exposed to high temperatures that make you perspire even when not working?” and “In your workplace, are you exposed to low temperatures, whether indoors or outdoors?” The participants could subjectively answer each question according to a seven-point scale (1 = all of the time, 2 = almost all of the time, 3 = around three-fourths of the time, 4 = around half of the time, 5 = around one-fourth of the time, 6 = almost never, and 7 = never). These scales were divided into three categories: never (almost never, never), moderate (around half of the time, around one-fourth of the time), and severe (all of the time, almost all of the time, around three-fourths of the time).

Injury experience in the workplace was determined in a similar manner. The question was: “Over the last 12 months, did you have any of the following health problems?” The participants responded “Yes” or “No” to the “Injuries” section. 

The PPE usage pattern was assessed through the following question: “Does your job ever require that you wear personal protective equipment (Safety helmet, safety gloves, masks, safety glasses)?” Participants who answered “no” were categorized as “No need.” Those who answered “yes” were asked the following additional question: “Do you always use it when it is required?” Those who answered “yes” to this question were classified in the group “Need/wear” and those who answered “no” were classified in the group “Need/no wear.” 

### 2.3. Covariates

Potential confounding variables, such as sex and age, were included as covariates. Socioeconomic factors, including education level and monthly household income, were also included, and classified into four groups. Other occupational characteristics included the following: working time (52 h or less and more than 52 h), shift work (yes or no), occupational classification (office, service and sales, manual workers), size of enterprise (fewer than 10 people, 10 to 49 people, 50 or more people), outdoor work (yes or no). Age was classified as 20–29, 30–39, 40–49, 50–59, 60–69, and 70–79 years. Occupational classifications were regrouped into three of ten major categories listed in the International Standard Classification of Occupations according to skills and duties: office (managers, professionals, technicians, and associate professionals), service and sales (clerks, service, sales), and manual workers (skilled agricultural and fishery, craft and related trades, plant and machine operators, assemblers, elementary workers).

### 2.4. Covariates

We used χ^2^ tests to compare the different characteristics and occupational factors of the participants according to injury experience. A binary logistic regression analysis was performed to investigate the association between the injury experience and high and low temperature exposures. The odds ratio (OR) and CI were calculated. Individual (e.g., sex, age, education level, and monthly income) and occupational factors (working time, shift work, occupational classification, size of enterprise, PPE usage, and outdoor work) were used as adjusted variables. Moreover, to identify the association between occurrences of injury from high and low temperature exposures owing to PPE usage, the participants were stratified according to the exposure of high and low temperatures, respectively. All statistical analyses were performed using SPSS for Windows version 18.0 (SPSS Inc., Chicago, IL, USA).

### 2.5. Ethics Statement

The KWCS questionnaire was collected after receiving written informed consent from all participants. Personally identifiable information was deleted before the data analysis. This study was a secondary data analysis, and approved by the Institutional Review Board (IRB) of Gachon Gil Hospital (IRB No. GFIRB2019-235).

## 3. Results

Table 1 presents the basic demographic characteristics of participants based on injury experience. The prevalence of injury experience was 2.1% (n = 963) for male workers and 1.3% (n = 600) for female workers. Statistically significant differences were observed between injury experience prevalence (yes or no), sex, age, education level, monthly income, working time, occupational classification, size of enterprise, high or low temperature exposure, PPE usage, and outdoor work experience (*p* < 0.05). Higher injury experience was associated with higher and lower temperature exposure, as well as participants who were older, had lower education levels, lower monthly income, longer working times, and smaller workplaces. If participants were manual workers, outdoor workers, or if PPE was needed but not worn, the injury experience was higher. Only shift work did not show statistically significant differences (*p* = 0.369).

The association between high and low temperature exposure, and injury experience, is shown in Table 2. The ORs and CIs were calculated using binary logistic regression analysis. The group that responded that they were not exposed to high or low temperatures served as a reference group.

### 3.1. High Temperature Group

For the high temperature exposure group, in model 0 (crude model), the OR for injury experience was 3.21 (95% CI = 2.87–3.60) for moderate exposure and 3.73 (95% CI = 3.26–4.26) for severe exposure. In model 1, where adjustment was made for individual factors (sex, age, education level, monthly income), the OR for injury experience was 2.59 (95% CI = 2.30–2.92) for moderate exposure and 2.76 (95% CI = 2.40–3.17) for severe exposure. In model 2, adjusted for both individual and occupational factors (working time, shift work, occupational classification, size of enterprise, PPE usage, and outdoor work), the OR for injury experience was 2.06 (95% CI = 1.78–2.38) for moderate and 2.15 (95% CI = 1.81–2.55) for severe exposure.

### 3.2. Low Temperature Group 

For the low temperature exposure group, in model 0, the OR for injury experience was 2.37 (95% CI = 2.10–2.67) for moderate and 2.19 (95% CI = 1.84–2.61) for severe exposure. In model 1, the OR for injury experience was 1.94 (95% CI = 1.72–2.19) for moderate and 1.72 (95% CI = 1.45–2.06) for severe exposure. In model 2, the OR for injury experience was 1.64 (95% CI = 1.44–1.85) for moderate and 1.39 (95% CI = 1.16–1.66) for severe exposure.

### 3.3. PPE Wearing Behavior and Injury Experience

In addition, to analyze the effects of PPE wearing behavior on the association between injury experience and exposure to high and low temperatures, participants were stratified according to PPE wearing behavior. For this analysis, the “no need” and “need/wear” groups were regrouped as the reference group, and a binary logistic regression analysis was performed on injury experience in the “need/no wear” group. This analysis was performed for both high and low temperature exposure groups. Moreover, the high and low exposure groups were regrouped as “no (“never”)” and “yes (“moderate” + “severe”).” The analysis was conducted only for groups with exposure.

Table 3 presents the effects of PPE wearing behavior by injury experience in the high and low temperature exposure groups. The adjusted variables are the same as in Table 2. For the high temperature exposure group, in models 0, 1, and 2, the ORs for injury experience were 1.64 (95% CI = 1.30–2.06), 1.52 (95% CI = 1.20–1.91), and 1.45 (95% CI = 1.15–1.83), respectively. For the low temperature exposure group, in models 0, 1, and 2, the ORs for injury experience were 1.58 (95% CI = 1.16–2.16), 1.35 (95% CI = 0.99–1.85), and 1.26 (95% CI = 0.92–1.73), respectively.

## 4. Discussions

The results of this study identified variables related to occupational injury in Korean workers. Through the χ^2^ tests, significant correlations were identified between occupational injury experience, personal characteristics (e.g., sex, age, education level, and monthly income) and occupational characteristics (e.g., occupational classification, size of enterprise, high temperature exposure, low temperature exposure, PPE wearing behavior, and outdoor work). These factors are reported to be associated with the occurrence of injury [21,24]. In addition, logistic regression analysis showed a significant correlation between high and low temperature exposures and injury experience even after correcting for these covariates.

Under heat stress in a hot environment, physiological changes occur, mental and physical task performances are reduced [26,27,28,29], as well as cognitive and reaction speed for risk [30,31], resulting in an increase in the accident rate [32,33]. There are also reports that grip problems can cause hand slippage [34]. This trend is mostly reported in the construction and agriculture industries, where there are many outdoor workers who are vulnerable to temperature control.

Meanwhile, in cold environments, agility is reduced; slippage and falls are more likely to occur [35] owing to limitations in movement by protective gear and reduced coordination capabilities [36]. In France, a questionnaire-based study [37] shows that injuries in cold environments are caused by environmental changes, such as snow, ice, and visual disturbance, and the incidence rate is three times that of a normal environment.

The damage caused by temperature change is more important to the socially and physically weak, such as older adults and workers in vulnerable environments. This is also an important concern when it comes to health inequality [16]. However, studies in developed countries have also reported more unintended injuries owing to changes in temperature [32].

The results of this study are summarized as follows: In the case of workers exposed to high temperatures, the group who had to wear PPE, but did not wear it, showed higher injury experience rates when compared with the reference group—even after adjusting for the covariates associated with injury occurrence. In contrast, for workers exposed to low temperatures, the correlation was not significant after the covariates were corrected.

A hot environment reduces the risk perception and risk response of workers, and affects irritability and aggression. It might be possible that body temperature will increase by PPE wearing [38]. A study analyzing Brazil’s tomato farms suggests that general attire takes 40 m to raise body temperature by 1 °C, but only 15 m under PPE—and the wearing rate of PPE decreases when the weather is hot [38]. The same study has pointed out that this tendency does not prevent the occurrence of neurotoxicity by chemicals, such as herbicides. PPE is thought to not only directly prevent the occurrence of injury in the event of an accident, such as falls and slips, but also affect indirect prevention, such as preventing the reduction of cognitive function from chemical neurotoxicity. Therefore, it is necessary to develop ergonomically designed PPE to overcome inadequate or uncomfortable PPE.

The present study has a number of strengths. The Korea Occupational Safety and Health Agency has systematically selected the KWCS population, representing Korean workers who work in hot and cold working environments. To the best of our knowledge, this is the first study to investigate the risks of injuries due to extreme temperatures in Korea. As workers are controlled by their employers, it is not easy to adapt or avoid climate change, so they need attention and protection [39]. We hope that these findings will promote future research regarding the health of workers who are working in low or hot temperatures—it is a topic that has been understudied thus far.

There was a limitation in this study, that high or low temperature exposure was the subjective answer of survey participants. It may have created possible bias, according to self-reported data on temperature exposure. Based on the results of this study, we believe that the effect of PPE wearing behavior on injury experience in high and low temperature environments can be more clearly identified in further study. However, prospective research, including quantitative assessment (measurement) of high and low temperatures, and specific medical assessment of injury, should be carried out by controlling for the type of PPE and its wearing behavior. In addition, ergonomic research is needed to develop PPE that can be comfortably worn in a high temperature environment, especially in response to, and in the context of, climate change. Above all, it is important to have workers wear PPE.

## 5. Conclusions

This study, using KWCS data, confirmed that exposure to high and low temperatures significantly increased the experience of occupational injury, even after correcting personal and occupational covariates related to occupational injury (OR for injury experience, high temperature exposure group = 2.15; low temperature exposure group = 1.39). In addition, we found that in the high temperature exposure group, “groups that require PPE but do not wear it” had more injury experience compared with the reference group, which is not required, and wear it (OR for injury experience, high temperature exposure but no wearing PPE = 1.45).

Further research to demonstrate the causal relationship between type of injury, extreme temperature, and ergonomics is necessary to develop PPE that can be worn comfortably in extreme environments.

## Figures and Tables

**Table 1:** Basic characteristics of study participants according to injury experience.

Characteristics	Total		Injury Experience				*p*-Value
			Yes		No		
	N	%	N	%	N	%	
Total	92,238	100.0	1563	1.7	90,675	98.3	
Sex							<0.001
Male	46,175	50.1	963	2.1	45,212	97.9	
Female	46,063	49.9	600	1.3	45,463	98.7	
Age (years)							<0.001
20–29	7769	8.4	68	0.9	7701	99.1	
30–39	16,648	18.0	193	1.2	16,455	98.8	
40–49	23,464	25.4	361	1.5	23,103	98.5	
50–59	23,720	25.7	437	1.8	23,283	98.2	
60–69	13,735	14.9	316	2.3	13,419	97.7	
70–79	6902	7.5	188	2.7	6714	97.3	
Education level							<0.001
Elementary school	8260	9.0	270	3.3	7990	96.7	
Middle school	8283	9.0	214	2.6	8069	97.4	
High school	36,612	39.7	696	1.9	35,916	98.1	
University or higher	39,083	42.4	383	1.0	38,700	99.0	
Monthly income (KRW)							<0.001
≤1 M	12,354	13.4	280	2.3	12,074	97.7	
≤2 M	27,297	29.6	419	1.5	26,878	98.5	
≤3 M	20,228	21.9	321	1.6	19,907	98.4	
>3 M	32,359	35.1	543	1.7	31,816	98.3	
Working time (h/week)							<0.001
≤52	79,747	86.5	1287	1.6	78,460	98.4	
>52	12,491	13.5	276	2.2	12,215	97.8	
Shift work							0.369
No	84,889	92.0	1448	1.7	83,441	98.3	
Yes	7349	8.0	115	1.6	7234	98.4	
Occupational classification							<0.001
Office workers	12,219	13.2	146	1.2	12,073	98.8	
Service and sales workers	49,191	53.3	549	1.1	48,642	98.9	
Manual workers	30,828	33.4	868	2.8	29,960	97.2	
Size of enterprise (number of employees)							<0.001
1–9	74,387	80.6	1283	1.9	67,379	98.1	
10–49	8771	9.5	134	1.5	8740	98.5	
>50	9080	9.8	49	1.2	4109	98.8	
High temperature exposure							<0.001
Never	68,527	74.3	729	1.1	67,798	98.9	
Moderate	15,481	16.8	517	3.3	14,964	96.7	
Severe	8230	8.9	317	3.9	7913	96.1	
Low temperature exposure							<0.001
Never	75,686	82.1	1044	1.4	74,642	98.6	
Moderate	11,504	12.5	369	3.2	11,135	96.8	
Severe	5048	5.5	150	3.0	4898	97.0	
Personal Protective Equipment							<0.001
No need	67,700	73.4	773	1.1	66,927	98.9	
Need/Wear	21,417	23.2	673	3.1	20,744	96.9	
Need/No wear	3121	3.4	117	3.7	3004	96.3	
Outdoor work							<0.001
No	78,235	84.8	1064	1.4	77,171	98.6	
Yes	14,003	15.2	499	3.6	13,504	96.4	

**Table 2:** Odds ratios and 95% confidence intervals for high/low temperature exposure by injury experience (binary logistic regression model).

Exposure	^b^ Model 0		*p* for Trend	^c^ Model 1		*p* for Trend	^d^ Model 2		*p* for Trend
	^a^ OR	95% CI		OR	95% CI		OR	95% CI	
High temperature									
None	1.00	ref.		1.00	ref.		1.00	ref.	
Moderate	3.21	2.87–3.60	<0.001	2.59	2.30–2.92	<0.001	2.06	1.78–2.38	<0.001
Severe	3.73	3.26–4.26		2.76	2.40–3.17	<0.001	2.15	1.81–2.55	
Low temperature									
None	1.00	ref.		1.00	ref.		1.00	ref.	
Moderate	2.37	2.10–2.67	<0.001	1.94	1.72–2.19	<0.001	1.64	1.44–1.85	<0.001
Severe	2.19	1.84–2.61		1.72	1.45–2.06	<0.001	1.39	1.16–1.66	

^a^ OR: odds ratio, CI: confidence interval, ref.: reference group. ^b^ Model 0: No covariates (crude); ^c^ Model 1: Individual factors (sex, age, education level, and monthly income); ^d^ Model 2: Model 1 + occupational factors (working time, shift work, occupational classification, size of enterprise, PPE usage, and outdoor work).

**Table 3:** Odds ratios and 95% confidence intervals for personal protective equipment wearing behavior by injury experience in the high and low temperature exposure groups (binary logistic regression model).

Exposure Group	PPE	^a^ Model 0		*p*-Value	^b^ Model 1		*p*-Value	^c^ Model 2		*p*-Value
		OR	95% CI		OR	95% CI		OR	95% CI	
High temperature	No need + Need/Wear	1.00	ref.		1.00	ref.		1.00	ref.	
	Need/No wear	1.64	1.30–2.06	<0.001	1.52	1.20–1.91	<0.001	1.45	1.15–1.83	<0.001
Low temperature	No need + Need/wear	1.00	ref.		1.00	ref.		1.00	ref.	
	Need/No wear	1.58	1.16–2.16	0.004	1.35	0.99–1.85	0.061	1.26	0.92–1.73	0.153

^a^ OR: odds ratio, CI: confidence interval, PPE: personal protective equipment, ref.: reference group; ^b^ Model 0: No covariates (crude); ^c^ Model 1: Individual factors (sex, age, education level, and monthly income); ^d^ Model 2: Model 1 + occupational factors (working time, shift work, occupational classification, size of enterprise, PPE usage, and outdoor work).
